# A Rare Cause of Neck Lump in an Infant

**DOI:** 10.24908/pocus.v7i2.15866

**Published:** 2022-11-21

**Authors:** David J McCreary, Salmah Lashhab

**Affiliations:** 1 Paediatric Emergency Department, Sunderland Royal Hospital, South Tyneside and Sunderland NHS Foundation Trust United Kingdom

**Keywords:** POCUS, paediatric POCUS, ewing sarcoma, neck mass, neck lump

## Abstract

A 5 month old girl presented to the Paediatric Emergency Department with a rapidly growing neck mass over 24 hours. She was systemically well and otherwise asymptomatic. On examination she had a mobile, soft and non-tender 5 cm x 5 cm neck mass. Blood tests were unremarkable with normal inflammatory markers. Point of Care Ultrasound (POCUS) was done which showed a solid left sided neck mass with increased vascularity but no evidence of collection or abscess. Given the atypical presentation and rapid growth the patient was commenced on empirical antibiotics and was discussed with both tertiary ENT and Oncology teams. An MRI was performed which was indeterminate. Biopsy of the neck mass was positive for Ewing Sarcoma. This is a rare case of Ewing Sarcoma in an infant. POCUS can be used to rule out common pathology and abnormal lymph nodes, aiding in ongoing investigation and management in neck lumps.

## Case

A 5 month old girl presented to the Paediatric Emergency Department (PED) after her parents noticed a large lump to the left side of her neck, seemingly appearing over the course of one day. The child was systemically well and had attended nursery the previous afternoon where it was not noticed by anyone. She had no fever, breathing difficulties or dysphagia and no restriction of neck movements or torticollis. A recent review of systemic symptoms was unremarkable apart from, on closer questioning, a cough which had been present intermittently over the past 6 weeks. The patient was born at term following an unremarkable pregnancy with normal antenatal scans. 

**Figure 1:**
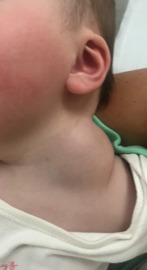
Large mass to left side of neck.

On examination a large mass was visible to the left side of her neck measuring approximately 5 cm x 5 cm (Figure 1). On palpation it was firm, not fluctuant and non-tender. There was no surrounding erythema or warmth. Ear, nose and throat examination was normal and she was apyrexial. 

Baseline blood tests were as follows; WBC 15.8 x 10'9 /L, Platelets 519 x 10'9 /L, Haemoglobin 125 g/L, Adjusted Calcium 2.70 mmol/L, Albumin 46 g/L, CRP 1.8 mg/L, and a normal coagulation screen. 

**Figure 2:**
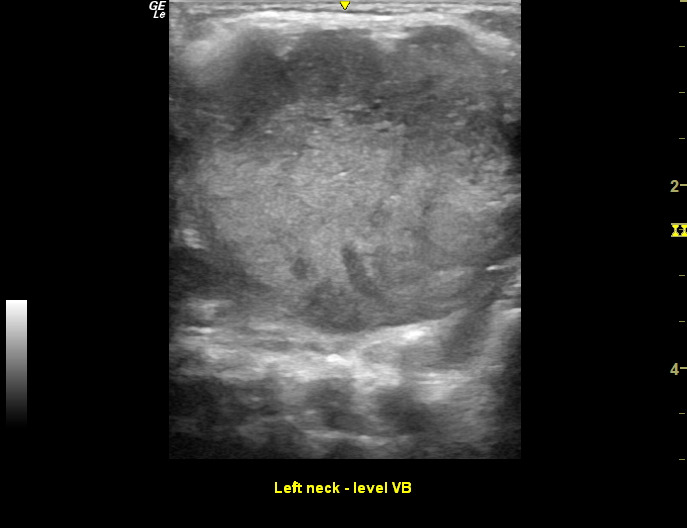
POCUS of neck mass level VB.

**Figure 3:**
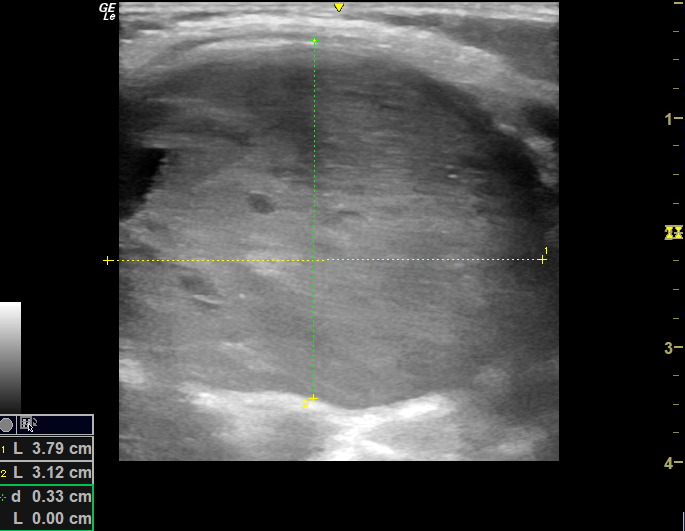
POCUS of neck mass.

Point of Care Ultrasound (POCUS) was performed using the linear, high-frequency 12 Mhz probe by one of the authors as shown in Figures 2 and 3. 

 

In the left neck level VB there was a large solid mass 3.4 cm x 4.1 cm. Rounded in shape with regular borders. Vascularity was increased. There was a maintained central echogenic hilum but also other abnormal increased echogenicity centrally. There was no fluid collection or evidence of abscess formation.

**Figure 4:**
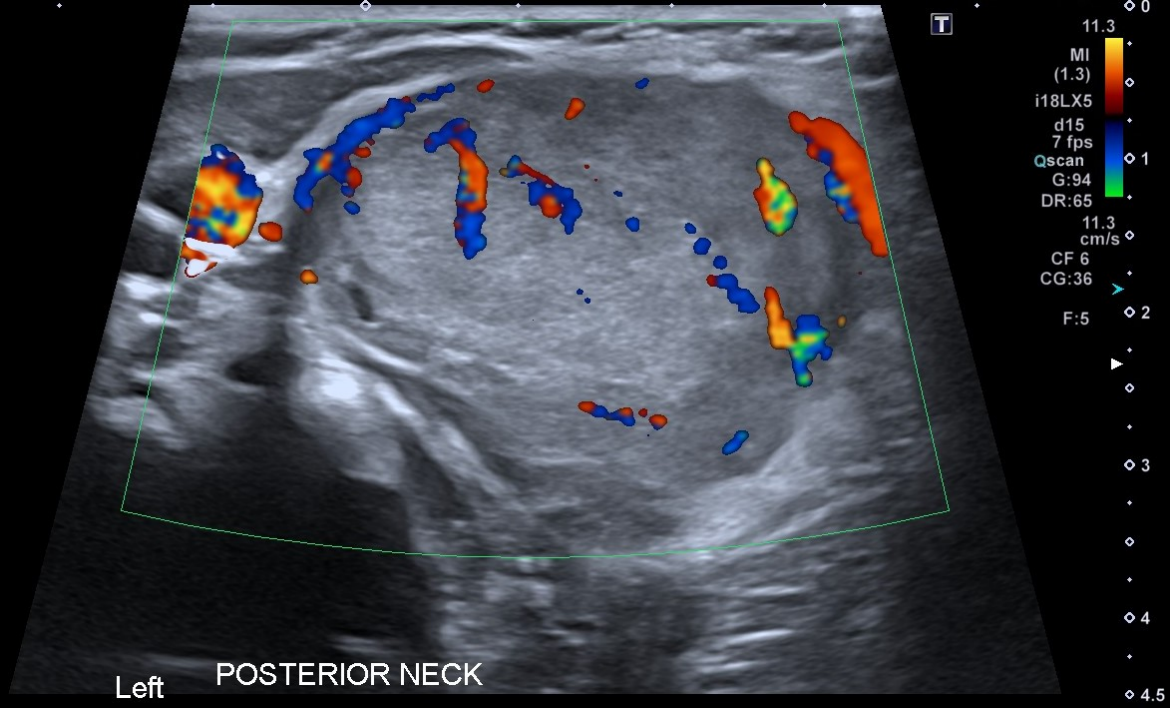
RADUS of neck mass.

Due to the appearances a formal radiology performed ultrasound (RADUS) was sought. This is shown in Figure 4. 

Differential diagnoses suggested by the radiology team included congenital cavernous haemangioma, vascular malformation, lymphangioma and dermoid cyst. 

A chest x-ray was performed to look for evidence of tuberculosis of which the child had no known contact. This was reported as normal with no lung lesions. The patient was commenced on IV Co-Amoxiclav and was reviewed by the local ENT team. Given the rapid evolution of the mass within 24 hours in an otherwise well child there was concern that the US report was not consistent with the clinical picture and therefore an MRI neck under sedation was planned. 

**Figure 5:**
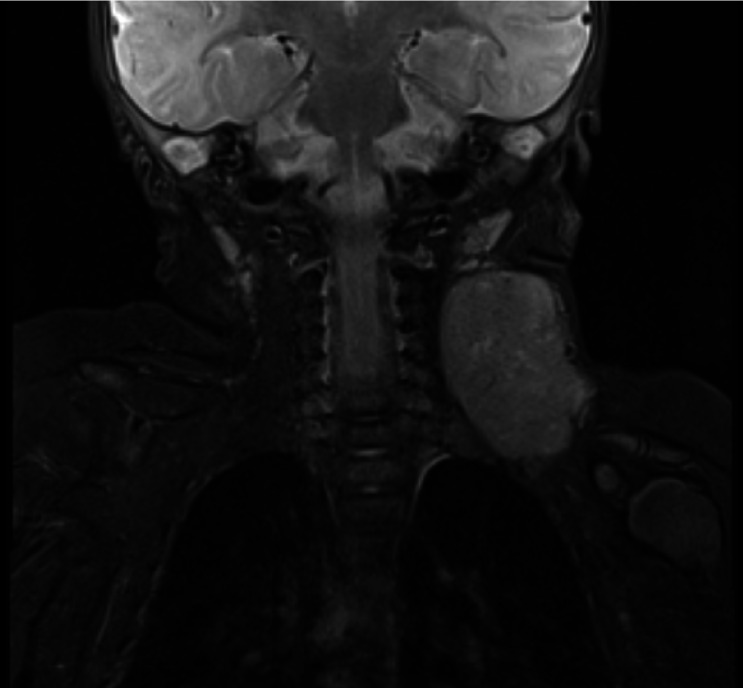
MRI soft tissue neck/thorax coronal plane.

**Figure 6:**
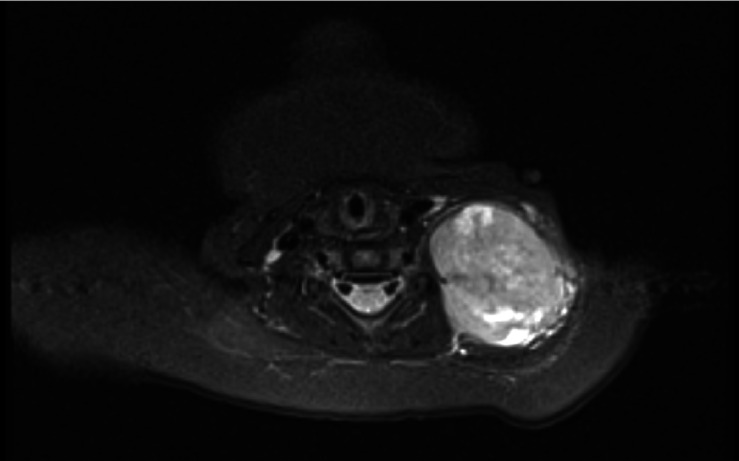
MRI soft tissue neck transverse plane.

The MRI report stated: a large well-defined soft tissue lesion is seen in left side of neck in levels III and IV/V 5 x 4.1 x 3.6 cm. Showing inhomogeneous low T1 and indeterminate T2 signal with diffuse post contrast enhancement. No significant inflammatory stranding. Nature of lesion - indeterminate - although sinister possibilities (e.g. sarcoma) should be considered. (See Figures 5 and 6 showing MRI images of neck lesion).

There was discussion regarding the nature of the mass between the local team and this was further discussed with the tertiary ENT and Oncology teams where it was agreed to transfer the patient for ongoing investigation and management. 

The mass was biopsied and histology showed Ewing Sarcoma. The patient underwent staging investigations and was planned to start chemotherapy. She she has since undergone Proton Beam therapy and is the youngest person in the United Kingdom to do so. 

This case demonstrates a very rare cause for a neck mass in an infant. Given the rapid evolution of the mass and age of the patient, use of POCUS was key in helping to identify concerning features associated with this mass and subsequently streamline further investigations and ongoing care. 

POCUS should be employed in order to answer binary questions which can help with hypothesis testing. In this case it was undertaken in order to differentiate less concerning swellings, such as a single or cluster of enlarged lymph nodes i.e. in lymphadenitis, from something more sinister. It was clear that this enlargement was not in keeping with a lymph node and although the appearances were not familiar to the operator, they were of sufficient concern that timely further investigation was sought. It was concluded that a radiology performed scan would be required urgently in order to stratify the risk associated with this potentially sinister mass. Clinical correlation prompted further action including specialty advice and further imaging.

### Use of POCUS to evaluate neck masses

In our PED POCUS is used by those with appropriate training to help differentiate abnormal lymph node enlargement, such as lymphadenitis and abscess formation, from that of simple reactive lymphadenopathy. 

Normal lymph nodes should be discrete, well-defined, hypoechoic bean or oval shaped structures. They should have distinct borders and a short axis-to-long axis ratio of less than 0.5. The central echogenic hilum should be readily appreciable 1.

A lymph node with an abscess, however, loses its architectural integrity often appearing more circular, occasionally with variable degrees of internal echoes and lacking internal vascular flow. 

**Figure 7:**
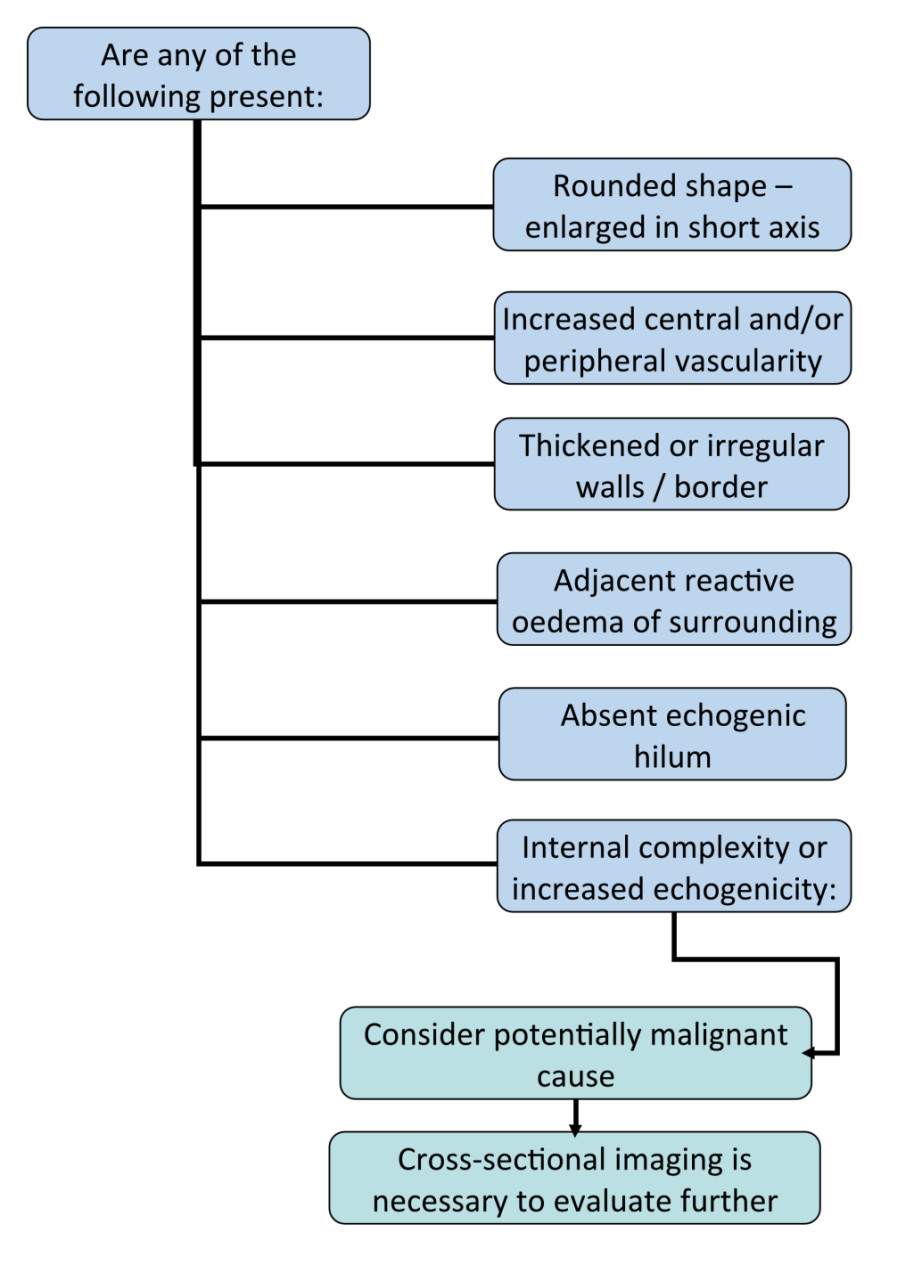
Example of POCUS algorithm for paediatric neck mass.

An enlarged lymph node with a more rounded appearance, i.e. an increase in diameter in the short axis, should be carefully interrogated. Thickened or irregular walls, loss of the normal echogenic hilum or internal features such as increased echogenicity (suggesting calcification, other infiltration or internal cystic change) should prompt the user to consider a potentially sinister cause such as the algorithm in Figure 7 demonstrates  2.

### Other neck masses encountered on POCUS

Simple cysts are mobile, often small and circular with thin walls with an anechoic centre. As they are entirely fluid-filled they demonstrate significant posterior acoustic enhancement. 

A sternocleidomastoid muscle tumour is another important differential for an infant with a neck lump which can lead to considerable distress for a parent. Fibromatosis coli can present within the early neonatal period as a firm, often large swelling within the territory of the sternocleidomastoid muscle and is entirely benign. It is most often related to birth trauma or instrumental delivery leading to fibrosis within the muscle. On POCUS this appears as fusiform enlargement of the sternocleidomastoid muscle belly where the muscle fibres can be seen running contiguous with the surrounding unaffected muscle fibres. There is no discrete mass observed 3.

Hemangiomas are congenital lesions made up of vascular tissue lined by endothelial cells. While often not present at birth they can grow rapidly within the first 3 months of life and become more prominent in size and number. Congenital cavernous haemangiomas are deeper and POCUS may reveal a cutaneous or subcutaneous soft tissue mass with prominent internal vascularity confirmed by enhanced colour flow 4.

Lymphangiomas are congenital abnormalities of the lymphatic system that most often affect the head and neck. Their classic appearances on ultrasound are that of multilocular cystic masses containing internal septa of varying thickness with occasional vascularity, though there is considerable heterogeneity 5. 

Salivary gland pathology, though relatively uncommon in children, is another group of diagnoses to consider when faced with an acute undifferentiated neck swelling. In acute sialadenitis, the inflamed affected gland appears enlarged, hypoechoic and hyperaemic on POCUS. Sialolithiasis, by contrast, demonstrates hyperechoic internal areas with distal acoustic shadowing 6.

Ectopic thymus can arise within the midline or lateral aspect of the neck and present as a mass in infancy or during childhood. Its characteristic thymic pattern provides the classical “speckled” appearances owing to the multiple, linear hyperechoic septa and homogenously distributed hyperechoic foci 3.

### Extraosseous Ewing sarcoma

Despite being the second most common primary sarcoma in children, Ewing Sarcoma is rare with only around 30 children in the UK diagnosed each year. There is a higher prevalence in males and adolescents 7. Ewing sarcoma commonly affects the bone (e.g. long bones, ribs, pelvis and spine) but can arise in soft tissue (extraosseous Ewing sarcoma) 8. The time from the initial onset of symptoms to diagnosis of Ewing sarcoma has a median interval reported from 2 to 5 months. They account for some of the most aggressive cancers with the potential for rapid metastasis and poor response to conventional chemo- and radiation therapy 9. In localised infiltration, chemotherapy has improved the 5-year overall survival rate from 10% to around 70% however primary metastatic disease treated with chemotherapy only has a 5-year overall survival of less than 30% 10. A definitive management plan for Ewing Sarcoma remains elusive. 

## Conclusion

POCUS is being increasingly utilised as part of the evaluation for the infant, child or young person presenting to the PED with an undifferentiated neck mass, as both a tool for aiding decision making and for streamlining patient flow. Studies have demonstrated the benefits it provides in terms of both diagnostic accuracy and an overall shorter length of stay in the PED 11, 12, 13, 14, 15.

## Disclosures

None.
